# Community Phylogenetics: Assessing Tree Reconstruction Methods and the Utility of DNA Barcodes

**DOI:** 10.1371/journal.pone.0126662

**Published:** 2015-06-25

**Authors:** Elizabeth E. Boyle, Sarah J. Adamowicz

**Affiliations:** Biodiversity Institute of Ontario & Department of Integrative Biology, University of Guelph, 50 Stone Rd. E., Guelph, Ontario, N1G 2W1, Canada; Institut National de la Recherche Agronomique (INRA), FRANCE

## Abstract

Studies examining phylogenetic community structure have become increasingly prevalent, yet little attention has been given to the influence of the input phylogeny on metrics that describe phylogenetic patterns of co-occurrence. Here, we examine the influence of branch length, tree reconstruction method, and amount of sequence data on measures of phylogenetic community structure, as well as the phylogenetic signal (Pagel’s λ) in morphological traits, using Trichoptera larval communities from Churchill, Manitoba, Canada. We find that model-based tree reconstruction methods and the use of a backbone family-level phylogeny improve estimations of phylogenetic community structure. In addition, trees built using the barcode region of cytochrome *c* oxidase subunit I (COI) alone accurately predict metrics of phylogenetic community structure obtained from a multi-gene phylogeny. Input tree did not alter overall conclusions drawn for phylogenetic signal, as significant phylogenetic structure was detected in two body size traits across input trees. As the discipline of community phylogenetics continues to expand, it is important to investigate the best approaches to accurately estimate patterns. Our results suggest that emerging large datasets of DNA barcode sequences provide a vast resource for studying the structure of biological communities.

## Introduction

The explicit application of phylogenetics to understanding community assembly was proposed by Webb [1,2], and community phylogenetics has since become a rapidly expanding field in ecology. The sorting of species is facilitated through environmental and biotic pressures, which can act at various phylogenetic and spatial scales [3]. Given that these different pressures leave distinct phylogenetic patterns between locally co-occurring species, we can distinguish between different processes of community assembly. Assuming phylogenetic niche conservatism, communities composed of closely related species (phylogenetically clustered) are typically interpreted as being primarily structured by an environmental filter, while communities containing distantly related species (phylogenetically overdispersed) are generally considered as indicating that competitive interactions are stronger in community assembly [1,2]. Mayfield and Levine [4] demonstrate how phylogenetic clustering may be caused by either environmental filtering or competitive exclusion, while overdispersion tends only to be associated with competition. Thus, while interpretation of patterns is not strictly dichotomous, phylogenetic community patterns provide important insight into community assembly, and this research area continues to grow [5].

Phylogenetic community studies determine the degree of phylogenetic clustering or overdispersion of co-occurring species. Metrics commonly applied that describe the phylogenetic community pattern are the net relatedness index (NRI) and the nearest taxon index (NTI) [1,2]. NRI refers to the standardized mean pairwise distance (MPD) between all pairings of co-occurring taxa, while NTI is the standardized version of the mean nearest taxon distance (MNTD) (i.e. the mean phylogenetic distance among just those pairings of co-occurring taxa that are the most closely related). NRI and NTI are standardized using the mean and standard deviation of null distributions of MPD and MNTD values, respectively, which are generated via random draws from the source phylogeny, keeping species richness constant and set to be equal to the richness in the observed community. This standardization enables NRI and NTI values to be compared across communities differing in richness. Increasingly positive values indicate phylogenetic clustering, and negative values indicate phylogenetic overdispersion. Because NRI incorporates the entire phylogeny into the calculation, while NTI is focused at the terminal branches [1], it is important to note that NRI and NTI can be informative of different patterns of co-occurrence on a phylogeny. For instance, communities may be comprised of multiple pairs or groups of closely related species, which would be indicated by a high NTI value, but across the phylogeny these tip clusters may be randomly distributed, which would lead to a NRI value nearer to zero.

The capabilities of these metrics to detect phylogenetic community structure and the factors that influence their power have been tested with regard to optimal model settings, phylogenetic scale, and geographic scale [3,6–9]. While these metrics have now become the standard for phylogenetic community structure studies, there has been little investigation into how these metrics are affected by the properties of the phylogenies used for generating them. Swenson [10] identified three phylogenetic issues that could potentially affect the power of the phylogenetic community structure metrics: (1) uncertainty and error in branch length estimates, (2) the assumption of correct topology, and finally (3) the presence of polytomies. Swenson [10] investigated the last of these and found that polytomies reduced the power of NRI and NTI to detect non-random communities (Type II error), and this was especially prevalent with deep polytomies in comparison to more terminal polytomies.

There has been further investigation into the effect of polytomies on metrics of phylogenetic community structure. More specifically, the use of plant DNA barcoding regions (rbcL, matK, and trnH-psbA) has been compared with results using less-resolved phylogenies constructed from Phylomatic [11]. Both studies found the Phylomatic phylogeny to have a higher incidence of being unable to detect non-random communities (i.e. higher Type II error) than the more resolved phylogenies [12,13]. These studies discuss the possibilities of using plant DNA barcode regions for phylogenetic community structure metrics; however, there has been no investigation of the applicability of animal DNA barcodes (the 5’ region of cytochrome *c* oxidase subunit I, COI [14]) to this field. Mitochondrial genes are expected, on average, to reconstruct less accurate relationships than nuclear genes at deeper nodes of a phylogeny due to higher rates of molecular evolution and saturation. In insects, mitochondrial genes have been found to have faster rates of evolution than nuclear genes, lower consistency index, higher base composition bias, higher transition:transversion ratios, and higher rate heterogeneity among sites, which suggest homoplasy [15]. However, previous research has found that there is comparable phylogenetic signal for resolving relationships amongst genera using COI compared to nuclear genes in Lepidoptera, but that the relative signal in COI declined at the sub-family and family levels [16]. It is possible that COI-based phylogenies may be most appropriate for calculating community structure metrics that focus along the tips of the phylogeny, at and below the genus level (i.e. NTI). However, the suitability of COI for these metrics needs to be tested to establish the power of using animal DNA barcode data for community phylogenetics studies.

As such, we currently have an inadequate understanding of: (1) how phylogenetic community structure metrics vary with differing branch length reconstructions; and (2) whether multi-gene data sets are significantly superior to single-gene phylogenies, specifically those constructed using the animal barcode region. If the phylogeny is biologically inaccurate with respect to branch length due to poor reconstruction methods (less input data, unrealistic substitution model), this may alter the community pattern detected. We hypothesize that COI has greater phylogenetic information for resolving more recent divergence events (e.g. intrageneric) compared to deep nodes, and therefore we predict that NTI calculations will be more accurate than NRI values when using a COI-based phylogeny. As well, with increased biological accuracy incorporated into the phylogeny construction, i.e. by using model-based phylogenetic methods, we would expect a better approximation of both the NRI and NTI values. Our study addresses the question of how choice of input phylogeny affects conclusions about phylogenetic community structure in a real field study.

To determine the processes dominating community assembly, it is also informative to assess the presence of phylogenetic signal in relevant trait data. For example, are related species significantly similar in traits such as body size, which are likely to be important for biological interactions? We therefore also assess the impact of input tree upon the two metrics of phylogenetic signal which are most commonly employed, Blomberg et al.’s K [17] and Pagel’s λ [18]. Blomberg et al.’s K < 1 suggests that traits display a lower phylogenetic signal than expected under Brownian motion, while K > 1 implies that traits display a stronger phylogenetic signal than expected (i.e. more strongly conserved) [17]. As with K, λ values near 0 imply no phylogenetic signal in the trait, and values close to 1 (or higher) indicate strong phylogenetic dependence of the trait [18].

To test these questions, we focused on real communities of Trichoptera larvae collected from Churchill, Manitoba, Canada. Trichoptera (the caddisflies) is a diverse and well-studied order of insects with well-supported phylogenies at the family level based on analysis of multiple genes and morphological characters [19,20]. In addition, a nearly comprehensive DNA barcode reference library has been constructed for the Trichoptera of the Churchill area [21–23], which has been made publically available on the Barcode of Life Data Systems (BOLD) [24]. This resource is a valuable aid for species-level identifications for the difficult-to-identify larvae [23]. In this study, we show that estimations of phylogenetic community structure using COI can be improved by using more phylogenetically robust reconstruction methods such as Bayesian inference and by incorporating a family-level backbone topology.

## Materials and Methods

### Field collection

We collected Trichoptera larval specimens from the subarctic location of Churchill, MB, Canada from June 5 to August 25, 2010. This research, which did not involve any human or other vertebrate individuals, embryos, or tissues, was conducted under a permit (WB11245) issued by the Manitoba Conservation Wildlife and Ecosystem Protection Branch from Winnipeg, MB to the Churchill Northern Studies Centre (CNSC) for conducting research in the Churchill Wildlife Management Area. We sampled specimens from a variety of freshwater habitats, including 30 rocky coastal bluff ponds, 30 tundra ponds, seven creeks, five lakes, and three points along the Churchill River (see Table A in S1 Table). Using a 250 μm dip net and hand picking, we sampled each location on three dates, once per month in approximately the same order. We defined a local community of potentially interacting individuals as the entire aquatic habitat encompassing both the benthic and pelagic regions. We standardized sampling effort across sites by the area sampled. For small ponds (<20 m of shoreline), we sampled the entire shoreline using the same protocol as for large habitats, executed to the degree possible given the nature of the habitat. If the habitat was large (i.e. a lake), then 20 m of shoreline was sampled. For large habitats we sampled a 20 m transect parallel to the shore, collecting 1 m away from the shore and then again 5 m from the shore or until a depth of 1.5 m. To ensure that all the species occurring in the location were collected, we performed the sweep along the transect twice in succession and retained a minimum of 10 individuals per field morphospecies at each site on each sampling date. Field morphospecies were delimited on site as individuals with similar size, colour, markings, and case material; these morphospecies were later validated through microscopic and genetic analysis. A sub-set of sites was sampled three times during one monthly visit; a comparison of genetically confirmed species accumulation curves for these sites verified that two sweeps accurately captured the local biodiversity [25]. We preserved specimens in 95% ethanol and upon return from the field stored them at -20^o^ C.

### Molecular analysis and species identification

We sequenced portions of one mitochondrial (COI) and three nuclear genes: cadherin (CAD), elongation factor 1 alpha (EF1-α), and RNA polymerase II (POL-II). These genes are commonly used for phylogeny reconstruction in Trichoptera [20,26,27], and we found high polymerase chain reaction (PCR) success based upon a pilot study including 2 other candidate gene regions (28S and Wingless). We sorted specimens to family based on Wiggins [28] and then, when available, selected 10 individuals per morphospecies (delineated using a microscope in the lab using the characters discussed in [23]) across all sites and samples for sequencing COI. We removed one leg from each specimen that we selected for sequencing and extracted the DNA using a standard, high-throughput invertebrate DNA extraction protocol [29]. We increased the initial DNA elution volume of 50 μl for COI to 100 μl for the nuclear genes as this increased amplification success.

To amplify the barcode region of COI, we performed PCR using standard DNA barcoding methods and a Lep/Folmer primer cocktail [30] (see Table B in S1 Table [27,31–34]). For the nuclear genes (CAD, EF1-α, and POL-II) (see specimen selection criteria below), we used a 25 μl reaction consisting of the same proportion of trehalose, 10x buffer, MgCl_2_, dNTP, and Platinum *Taq* polymerase as used for COI, but we increased the amount of forward and reverse primer to 1.25 μl of 10 μM and added 2 μl of DNA template. Successful PCR products were bidirectionally sequenced using protocols outlined by the Canadian Centre for DNA Barcoding [35] (see Table C in S1 Table).

Using Codon Code Aligner (Codon Code Corporation, v. 3.7.1), we edited and concatenated the forward and reverse chromatograms. We then subsequently aligned sequences using Clustal W and checked the amino acid sequences for all genes for stop codons and indels in MEGA 5.0 [36]. We uploaded sequences to BOLD and identified the specimens to the species level using only the expert-identified published database of COI sequences available on BOLD that had ≥98% sequence similarity to our sequences [21,23]. We employed a 2% threshold for sequence dissimilarity since previous work has found this cut-off to correspond closely to morphological species limits, as determined using both adults and larvae, for Trichoptera species of the Churchill region [21–23]. For completing the species presence/absence matrix, we performed additional COI sequencing to obtain species-level identifications for those taxonomic groups where our prior morphospecies designations did not perfectly match the COI genetic clusters.

After completing the COI work, we selected one specimen per species for the nuclear gene sequencing; we randomly selected an individual among those having a high-quality COI sequence (658 base pairs [bp], 0 ambiguous bases) that was closely related to the other specimens of that same species (i.e. not an outlier for its species on the neighbour-joining phenogram). Sequences of the nuclear genes were verified by using BLAST on GenBank and by building single-gene phylogenies to screen for contaminants, in addition to inspecting the amino acid alignment. All of our sequences are currently stored on BOLD in the public project EBTCH, Trichoptera Larvae of Churchill 2010 and on GenBank (COI: JX681817—JX682383; CAD: KR030383—KR030415; EF1-α: KR030340—KR030382; POL-II: KR271613-KR271655) (see Table D in S1 Table).

As Lepidoptera is the well-supported sister group to Trichoptera [37], we rooted all trees using sequences for Lepidoptera species downloaded from GenBank (GenBank IDs: *Coleophora serratella* COI—GU828594.1, CAD—GU828096.1, and EF1-α - GU828929.1; *Phalera bucephala* COI- GU828607.1, CAD—GU828108.1, and EF1-α - GU828941.1; *Hypenodes humidalis* COI—GU828672.1). No lepidopteran sequences for POL-II were available at the time of analysis for these species.

### Phylogeny construction

To assess the influence of phylogenetic reconstruction on community structure metrics, we built in total six species-level phylogenies based upon a single individual per species: (1) multi-gene Bayesian; (2) COI Bayesian; (3) COI Bayesian phylogeny with a constrained backbone topology at the family level based on Holzenthal et al. [19]; (4) COI Bayesian with no backbone but with family-level branch lengths stretched 2x their original length and (5) 5x their original length, to test whether a simplistic correction for transitional saturation may result in COI-based trees yielding similar results as obtained using multi-gene nuclear phylogenies [16]; and finally (6) COI Neighbour Joining. Finally, as a negative control to examine metric behaviour, we also built a random tree by using the COI Bayesian phylogeny for the topology and branch lengths and then randomly shuffling the taxa among the tips in Mesquite 2.75 [38].

For the construction of the Bayesian phylogenies, we selected the best model of nucleotide substitution based upon the lowest *Akaike information criterion* (AIC) score for each gene using MrModelTest 2.3 [39], in conjunction with PAUP 4.0 [40]. For all genes, MrModelTest found the best model to be the generalized time reversible (GTR) with a gamma distribution parameter describing among-site rate variation and a proportion of invariant sites parameter. We built all Bayesian phylogenies in MrBayes 3.2 [41] using 4 chains with 10,000,000 generations, a sampling and diagnostic frequency of 1000, and a 25% burnin.

For the construction of the COI Neighbor Joining (NJ) tree, we used MEGA 5.0 to select the best model of nucleotide substitution and build the tree. A Tamura-Nei model with a gamma distribution was applied, and a bootstrap test with 1000 replicates was performed to assess the support for the tree.

### Congruence of distance matrices

Since all phylogenies are converted to species pairwise distance matrices for calculating the phylogenetic community metrics, we first evaluated how different the input matrices were from one another by calculating the congruence among distance matrices (CADM) metric in R using the package ape [42–44]. We performed *a posteriori* testing to assess which matrices are the most incongruent.

### Phylogenetic community structure metrics

For each of the 6 phylogenies, we calculated NRI and NTI. We calculated each metric using the same observed species presence/absence matrix created for local communities determined for Trichoptera larvae for the Churchill area from the DNA-barcode-validated morphospecies (see Table E in S1 Table). Each timepoint (June, July, August) for which two or more species were detected within a site was treated as a separate community, for a total sample size of 101 communities analyzed (see Table E in S1 Table). As this study is investigating impact of molecular dataset and phylogenetic analysis methods upon community phylogenetic metrics, no temporal analysis was performed here (but see [25]). In the Picante package in R, we calculated NRI and NTI using a null model with 1000 randomizations and 1000 iterations using the independent swap algorithm [45].

We used our multi-gene Bayesian phylogeny as the default against which to compare the community structure metrics generated using the other phylogenies. Although our goal is to assess the impact of input phylogeny upon conclusions from a real field study, not to assess which phylogeny is most likely to represent the truth, we assumed for our purposes here that the multi-gene Bayesian tree would be the strongest phylogenetic hypothesis, and therefore the most accurate for estimating metrics of phylogenetic community structure. Increased phylogenetic accuracy is usually achieved by increasing the number of independent genes and by utilizing more complex models of evolution [46–48]. We therefore performed a linear regression of the multi-gene Bayesian-calculated NRI and NTI values against those from all other phylogenetic hypotheses in sequence, with the origin forced through zero, as in Swenson [10], using R. A slope close to 1 would indicate that the values estimated by the other phylogenies are very similar to those estimated by the multi-gene Bayesian phylogeny, while a high r^2^ would suggest that there is little variation between the values calculated by the other phylogenies vs. the multi-gene Bayesian phylogeny.

### Phylogenetic signal metrics for traits

To test the influence of phylogeny on phylogenetic signal metrics for traits, we calculated two metrics prevalent in the literature, Blomberg et al.’s K statistic [17] and Pagel’s λ [18], for maximum body length and maximum case length (see Table F in S1 Table). Body size of Trichoptera larvae has been linked to habitat preferences [49,50] and is a commonly used ecological trait to test for phylogenetic signal in a variety of taxonomic groups [51]. Using a Nikon AZ100M microscope and NIS elements BR 3.0, we measured body length (mm) from the tip of the mandible to the end of the anal claw on specimens, while case length (mm) was the maximum distance spanned. After an initial visual sort of the largest specimens of each COI-identified species, microscopic measurements were taken on one to eight specimens, with the sample size dependent on abundance, enabling the measurement of maximum size for each species among the specimens we processed. Four species of Trichoptera (*Hydropsyche alternans*, *Neureclipsis crepuscularis*, *Polycentropus aureoles*, and *Rhyacophila angelita*) were excluded from the case length analysis, as they do not build portable cases, but either make fixed retreats or are free living [28].

We utilized the maximum length measured for each species and calculated Blomberg et al.’s [17] K statistic with 1000 replicates using the package Picante in R [45]. In addition, we calculated Pagel’s λ [18] using Geiger in R [52] and used a likelihood ratio test and a chi-squared distribution to compare the estimated λ to a tree with no phylogenetic signal (λ = 0), thus testing for significant phylogenetic structure in the trait data.

## Results

**Fig 1:**
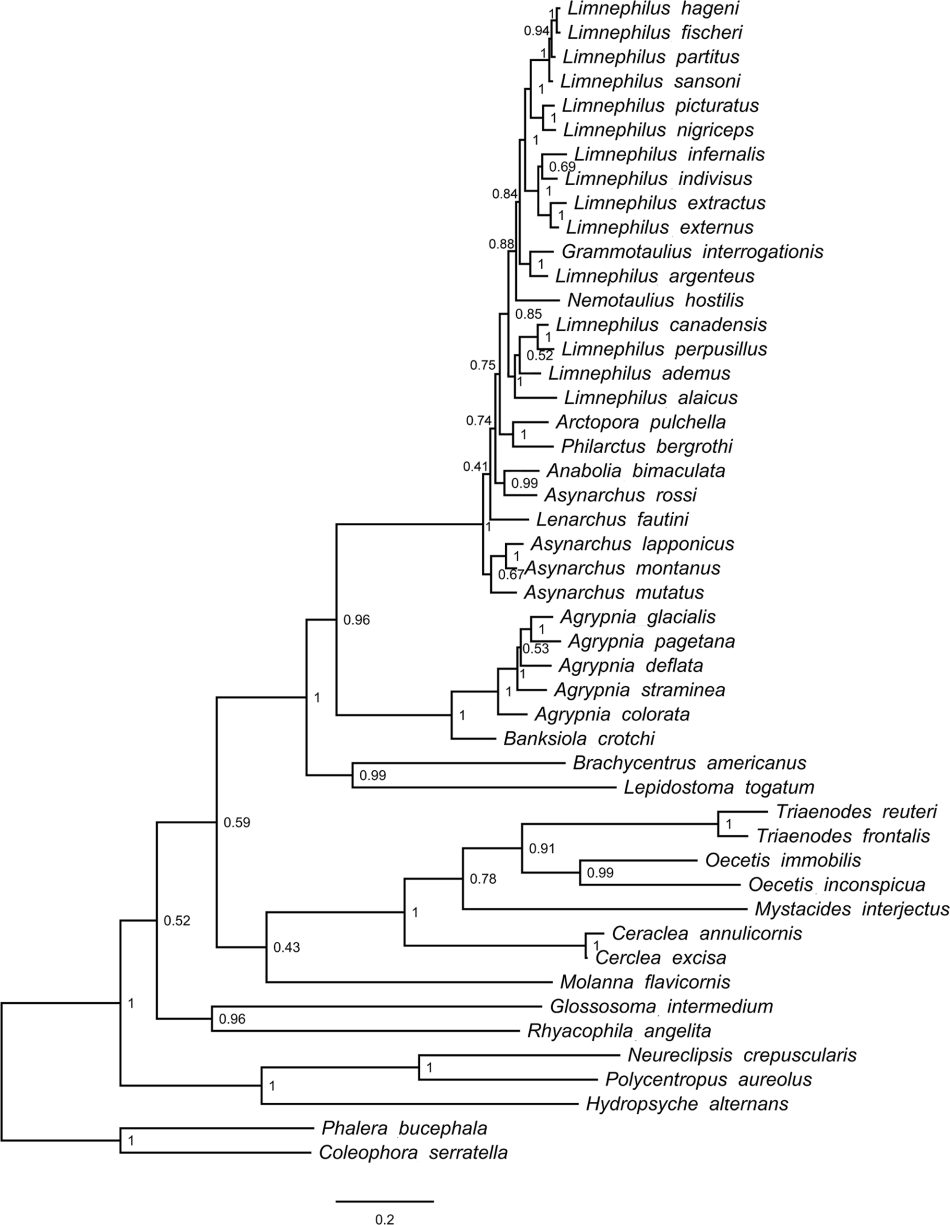
Trichoptera Bayesian tree built with COI, CAD, EF1-α, and POL-II. Node values indicate estimated posterior probabilities from Bayesian analysis.

**Fig 2:**
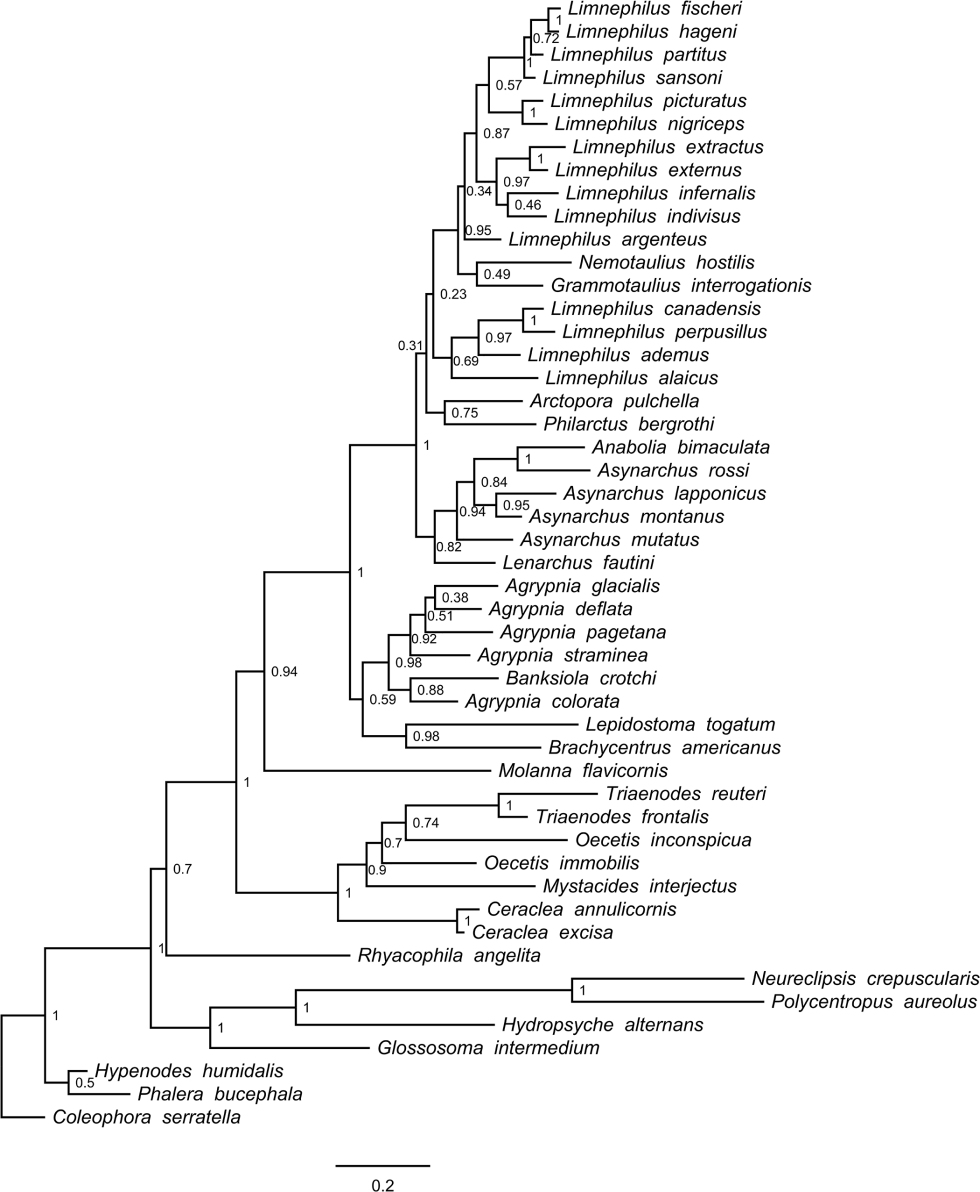
Trichoptera Bayesian tree built with COI. Node values indicate estimated posterior probabilities from Bayesian analysis.

**Fig 3:**
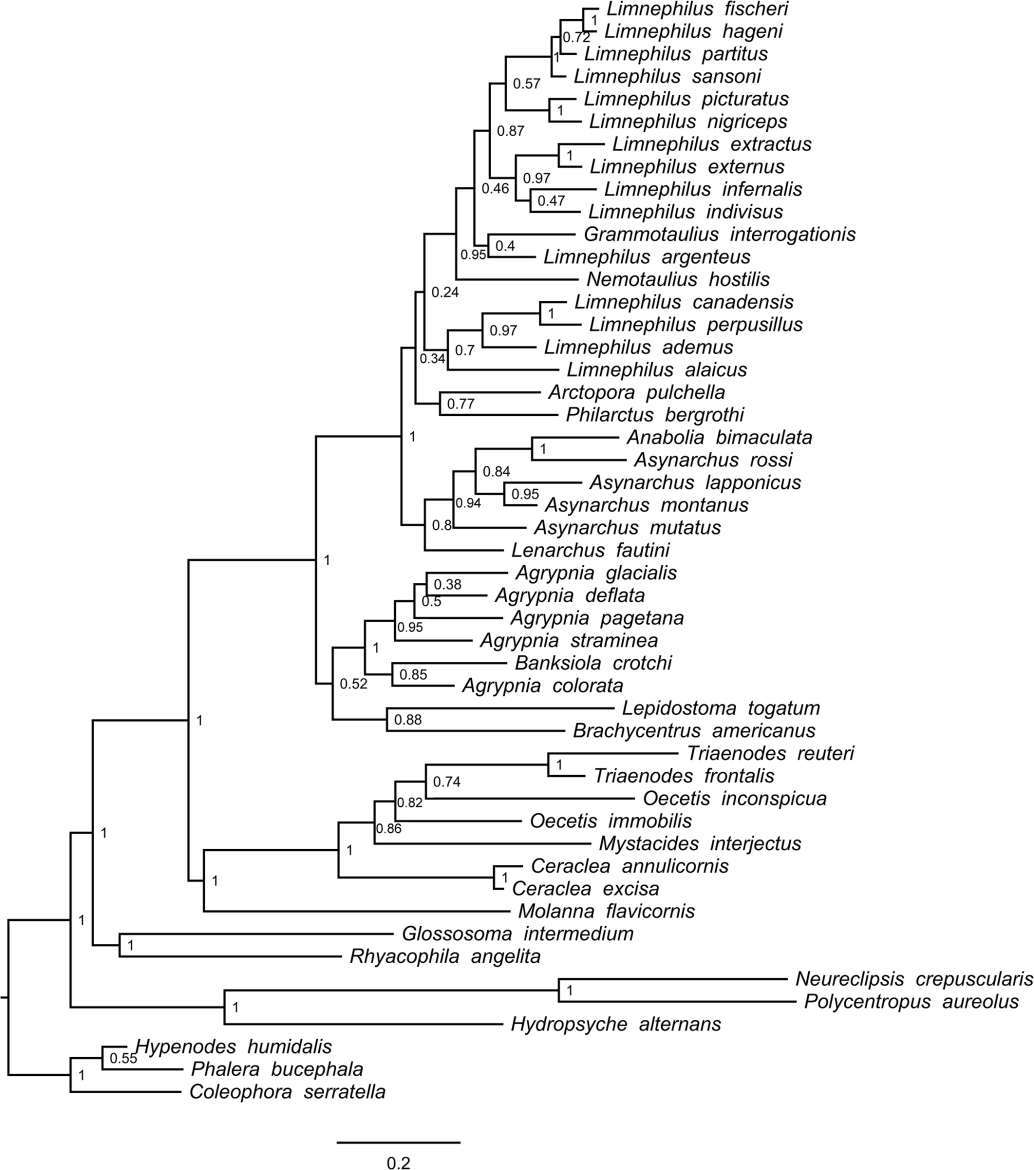
Trichoptera Bayesian tree built with COI and a backbone phylogeny enforced, using family relationships from Holzenthal et al. [19]. Node values indicate estimated posterior probabilities from Bayesian analysis.

**Fig 4:**
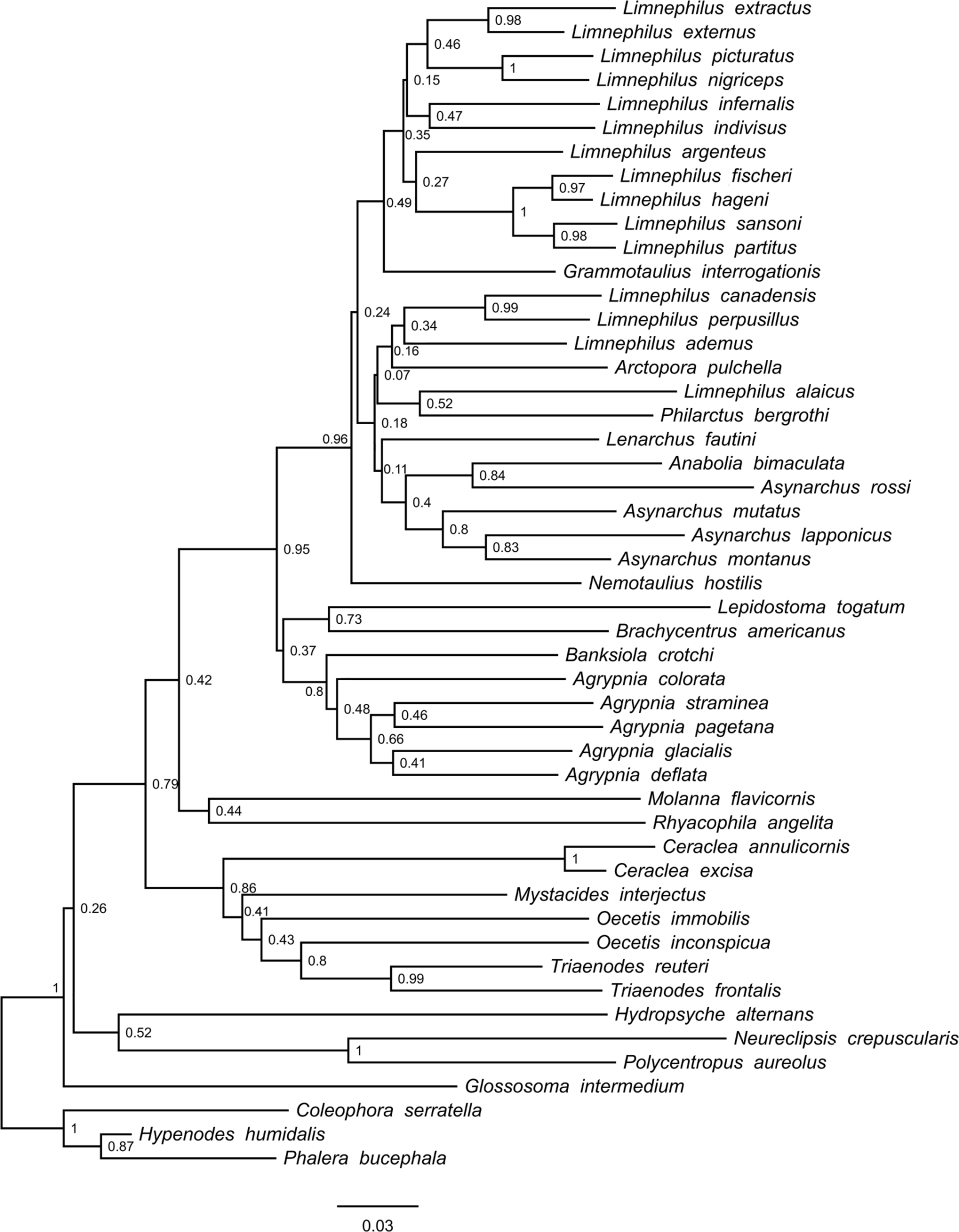
Trichoptera Neighbour Joining tree built using COI. Node values indicate bootstrap values.

In total, we processed 570 Trichoptera larval specimens for the COI molecular analysis, of which 99.5% produced a successful sequence. We found 46 species among our samples from the Churchill region. Of these, we were able to recover sequences for 43 species for POL-II (93%), 43 species for EF1-α (93%), and 33 species for CAD (72%). After trimming sequences so that at least 50% of species were represented at the beginning and the end of the alignment, COI consisted of 658 bp, POL-II 712 bp, EF1-α 483 bp, and CAD 730 bp. None of the alignments contained any stop codons or gaps. In general, our original morphospecies identifications, as defined using characters discussed in Ruiter et al. [23], were well supported by the COI genetic clusters. Figs 1–4 present the multi-gene Bayesian phylogeny, COI Bayesian phylogeny, COI Bayesian + backbone phylogeny, and COI NJ tree, respectively.

### Distance matrix similarity

The distance matrices from all of the phylogenies (exempting the random phylogeny) were congruent. *A posteriori* testing showed that the mean of the Mantel correlations, computed on rank-transformed distances, between the multi-gene Bayesian distance matrix and all others were high (>0.9, p = 0.010 for all trees; Table 1). The trees with the highest to lowest congruence score, as compared with the multi-gene Bayesian tree, were: COI Bayesian + backbone > COI Bayesian > COI Bayesian 2x > COI Bayesian 5x > COI NJ.

**Table 1:** Mantel correlations and probabilities of genetic distance matrices of phylogenies in comparison to the multi-gene Bayesian phylogeny.

Phylogeny	Correlation	Probability
COI Bayesian	0.968	0.010*
+ backbone	0.970	0.010*
stretched 2x	0.964	0.010*
stretched 5x	0.957	0.010*
COI NJ	0.917	0.010*
Random	-0.068	0.693

* Significant at p < 0.05; the null hypothesis of incongruence is rejected.

Probability values given for the congruence among distance matrix (CADM) test, which uses a null hypothesis of incongruence.

### Estimation of phylogenetic community structure metrics

NRI and NTI values calculated using the multi-gene Bayesian phylogeny distance matrix were well estimated using the other phylogenies, excluding the random phylogeny (Table 2). We observed a strong association between the NRI and NTI values of the multi-gene phylogeny and NRI and NTI values for all other phylogenetic hypotheses (r^2^>0.75), and the slopes were slightly below 1.

**Table 2:** Linear regression of phylogenetic community metrics using the multi-gene Bayesian phylogeny against those based on other phylogenetic hypotheses.

	NRI		NTI	
	Slope	r^2^	Slope	r^2^
COI Bayesian	0.937	0.874	0.898	0.862
+ backbone	0.945	0.898	0.893	0.878
stretched 2x	0.964	0.869	0.894	0.841
stretched 5x	0.987	0.848	0.903	0.787
COI NJ	0.897	0.894	0.817	0.838

Both the NRI and the NTI values were well estimated by the COI Bayesian + backbone phylogeny. The COI Bayesian + backbone phylogeny yielded NRI and NTI values that displayed the highest r^2^ when regressed against the metrics as obtained using the multi-gene Bayesian phylogeny (0.898 and 0.878, respectively); slopes were close to 1 (0.945 and 0.893, respectively). The COI Bayesian 5x performed best in terms of the closest slope to 1 (NRI = 0.987, NTI = 0.903); however, it had a lower r^2^ value than several other phylogenies (NRI = 0.848, NTI = 0.787).

### Presence of phylogenetic signal

For body length, K showed significant phylogenetic signal for all of the phylogenies except the multi-gene Bayesian phylogeny (Table 3). Case length was shown to display phylogenetic signal based on K for only the NJ tree (p = 0.018). By contrast, λ detected significant phylogenetic signal across all of the phylogenies for both body length and case length (p<0.05). For both traits, the lowest λ values were calculated from the multi-gene Bayesian phylogeny (0.316 for body length, 0.308 for case length), while λ was somewhat higher for the other phylogenies and highest using the NJ tree (0.532 for body length, 0.581 for case length).

**Table 3:** Phylogenetic signal metrics for Trichoptera maximum body length and maximum case length, using Blomberg et al.’s K metric [17] and Pagel’s λ [18].

	Body Length				Case Length			
Phylogeny	K	p-value	λ	p-value	K	p-value	λ	p-value
Multi-gene Bayesian	0.116	0.142	0.316	0.013*	0.150	0.191	0.308	0.044*
COI Bayesian	0.344	0.026*	0.450	0.009*	0.455	0.061	0.516	0.004*
+ backbone	0.327	0.043*	0.438	0.010*	0.445	0.062	0.509	0.004*
stretched 2x	0.210	0.034*	0.469	0.005*	0.296	0.067	0.514	0.003*
stretched 5x	0.092	0.032*	0.510	0.004*	0.139	0.054	0.490	0.003*
COI NJ	0.809	0.007*	0.532	0.018*	0.923	0.018*	0.581	0.008*

* Significant at p < 0.05.

## Discussion

While studies on phylogenetic community structure have provided exciting insight into community assembly, it is important to evaluate the methods applied. Although the power, taxonomic scale, and spatial scale of phylogenetic community structure metrics and null models have been investigated [3,6–9,53], both the theoretical underpinnings of this approach (e.g. Mayfield and Levine [4]) as well as the behaviour of key test statistics require further study. Our study has focused on examining several of the phylogenetic problems proposed by Swenson [10], which have received little attention to date in the literature.

### Best approaches for estimating phylogenetic community structure

Our study investigated how different phylogenetic reconstruction methods applied to COI data can approximate NRI and NTI calculated from a multi-gene tree, presumed here to provide the more robust phylogenetic hypothesis and branch lengths across the entire depth of the phylogeny. We found that both NRI and NTI values calculated from COI Bayesian phylogenies were generally concordant with those generated using the multi-gene tree, therefore not supporting our original hypothesis. Since the COI distance matrices were congruent with the multi-gene genetic distance matrix, and the phylogeny was generally well supported at the deeper nodes, it is not surprising that NRI and NTI values were also well estimated. Therefore, the rather surprising finding from our study was how well the COI data estimated the relative genetic distances between pairs of co-occurring species. All COI Bayesian phylogenies and the COI NJ tree had slopes slightly less than 1 for both NRI and NTI, indicating a slight bias towards overestimating the probable true value, i.e. increased detection of non-random phylogenetic community structure (Type I error). It is also interesting to note that the estimated NRI and NTI have a higher variance for values indicative of overdispersed phylogenetic community structure than for clustered values (S1 Fig and S2 Fig). This variance may be the result of the branch lengths being more poorly estimated for deeper nodes of the phylogeny for COI.

In terms of which phylogenetic reconstruction method most accurately estimated the multi-gene phylogenetic community structure metrics, we found that a Bayesian approach using COI while enforcing a backbone for the deeper relationships in the phylogeny performed well. The COI Bayesian + backbone phylogeny had the highest r^2^ values for both NRI and NTI when comparing against the multi-gene Bayesian tree, indicating that this approach yielded values that varied little from the multi-gene Bayesian NRI and NTI values. In addition, the COI Bayesian + backbone phylogeny for NRI and NTI had slopes close to 1, suggesting that the values estimated using the COI Bayesian + backbone were very similar to the multi-gene Bayesian phylogeny. It is intuitive that this approach produced the most accurate NRI and NTI values, as it also produced the most congruent distance matrix with the multi-gene phylogeny. In addition, it was expected that a stronger phylogenetic reconstruction method including more biological information and a more realistic model of molecular evolution would produce a more accurate phylogeny and thus estimates of phylogenetic community structure metrics. As such, we would recommend that future community phylogenetic studies use realistic phylogeny reconstruction methods, as well as include information about the supported relationships among taxa from prior studies, particularly when using animal DNA barcode data for community phylogenetics.

### Discrepancy of metrics measuring phylogenetic signal

Our study revealed substantial variation between the two metrics measuring phylogenetic signal in trait data but limited variability in conclusions across the phylogenetic reconstruction method employed. Similar to Münkemüller et al. [53], we found that Blomberg et al.’s K and Pagel’s λ suggested different conclusions about the phylogenetic signal of traits in our phylogenies. For instance, K suggested that both traits are not significantly conserved in the multi-gene Bayesian phylogeny; however, λ implied that they are significantly conserved. For all of the COI Bayesian phylogenies for case length, K found no support for phylogenetic signal, while λ found significant phylogenetic signal.

Münkemüller et al. [53] used simulated data to investigate the sensitivity of these metrics to phylogenetic structure and found that λ had similar values with repeated simulations, was less sensitive to variation in the number of species in the phylogeny, and was less prone to missing branch length information than K. In addition, λ had a smaller Type I statistical error rate and was able to detect phylogenetic signal in traits evolving under Brownian motion better than K, which was prone to Type II error [53]. Freckleton et al. [51] also reported appropriate Type I error rates for λ in simulated data and found that λ was able to detect phylogenetic signal in >90% of simulations for trees with 20 species and close to 100% for trees with 40 species. In addition, Freckleton et al. [51] found that 88% of published phylogenies display phylogenetic signal for at least one morphological or ecological trait with λ, and overall, 60% of traits displayed phylogenetic signal.

In our study, the K metric is failing to detect phylogenetic signal in the traits we measured, but λ suggests that phylogenetic dependence is present. Our phylogenies contain a reasonable sample size of species for this type of study (n > 40), as indicated by simulations and a review of λ values from empirical studies of morphological and ecological traits; therefore, our study should have strong power to detect significant phylogenetic signal, if present, using λ [51]. Since we do not explicitly know the process of trait evolution for our Trichoptera phylogenies, we cannot state which metric is superior to the other. However, we tend to favour Pagel’s λ for assessing phylogenetic dependence of trait data due to prior evidence of this metric’s behaviour [53], as well as the frequent finding of phylogenetic signal in body size measures across diverse taxa [51]. Focusing on our λ results, conclusions about the significance of phylogenetic signal did not vary across input trees; however, all of the COI phylogenies slightly overestimate phylogenetic signal when compared with the multi-gene tree. In sum, we conclude that choice of input tree was of modest impact in our study, when using λ, but that the Bayesian COI trees recovered λ values closer to those for the multi-gene phylogeny than did COI NJ trees.

### Utility of COI

Our study has highlighted the utility of COI for estimating phylogenetic community structure for local communities within a small regional source pool within the order Trichoptera. Of course, when more genetic information is available, it is expected to be useful for better estimating NRI and NTI; nonetheless, our results suggest that using only COI is a reasonable approach at the taxonomic and geographic scale examined. We stress that for calculations of phylogenetic community structure, branching order is not as important as relative branch lengths within a dataset, as the tree is converted to a distance matrix for calculations. Short internal branches may be associated with rapid successive diversification events; different phylogenetic reconstructions may yield different branching orders yet similar relative values for the total sums of the branch lengths connecting pairs of tips. Therefore, the main consideration for community phylogenetics is being able to accurately estimate the relative genetic distances between species, which COI appears to do quite well at the geographic and taxonomic scale examined. However, for phylogenies reconstructed using only COI, theoretically we expected NTI to be more accurately estimated than NRI, since NTI focuses on the tips of the phylogeny where there is expected to be more support for a COI phylogeny. By contrast, our results indicated that NRI had higher r^2^ values and slopes closer to 1 (in comparison with NRI from the the multi-gene tree) than NTI values. However, this finding may be an artefact of the communities in Churchill being principally phylogenetically clustered; the performance of NRI may be reduced when the communities are predominantly overdispersed, and this is a subject requiring further investigation. The Churchill area that we sampled is classified as a region according to Webb et al. (10–1,000 km) [2], and while our study suggests that COI is suitable at this scale for estimating NRI and NTI, difficulties may arise when examining larger geographic areas and broader taxonomic groups. It might be expected that a wider geographic area would include more taxa (species-area relationship; [54]), which is expected to increase phylogenetic accuracy. However, at a broader geographic scale, more taxonomic families may be sampled, yielding more deep branches requiring resolution, which could reduce the accuracy of a COI phylogeny and thus the NRI and NTI of the community. Nevertheless, improved phylogeny reconstruction methods are allowing researchers to build accurate phylogenies amongst insect orders with mitochondrial genomes [55]; with improved analytical methods, together with the availability of more backbone phylogenies, these problems may be alleviated. In addition, the accuracy of specimen identifications using COI may be reduced at broad vs. local geographic scales in some taxa [56]; hence, this should be another consideration for future studies. Despite these challenges, the large quantities of geo-referenced DNA barcodes being generated from initiatives such as the International Barcode of Life (iBOL) project (www.ibol.org) could be a vast, largely untapped resource of community data at varying geographic scales.

## Conclusions

Understanding the most robust approaches to addressing questions of community phylogenetics is critical to ensure that meaningful conclusions are drawn about the mechanisms driving community assembly. Our study has examined several different techniques of tree reconstruction and identified the strongest methods and metrics for our study system, which future researchers may consider in their study design. Since phylogenetic community ecology is a rapidly expanding field that holds much potential for understanding community structure, it is important to couple observational research with a broader understanding of the methods employed in the field.

## Supporting Information

S1 Fig Linear regression of the NRI values for Churchill Trichoptera larval communities calculated using the multi-gene Bayesian phylogeny against those calculated using other phylogenetic hypotheses. Red lines show the equation for each linear model, which is forced through 0.
(TIFF)

S2 Fig Linear regression of the NTI values calculated using the multi-gene Bayesian phylogeny against the NTI values generated using other phylogenetic hypotheses. Red lines show the equation for each linear model, which is forced through 0.
(TIFF)

S1 Table Supporting Tables. Table A. Site information for Trichoptera larval communities collected from Churchill, Manitoba, Canada. Table B. List of primers used in this study. Table C. List of thermocycling regimes used in this study. Table D. List of sequences and accession numbers for Trichoptera larvae of Churchill, Manitoba, Canada. Table E. Species presence/absence matrix for Trichoptera larval communities of Churchill. Table F. Morphological measurements for Trichoptera of Churchill.
(XLS)

## References

[1] Webb CO (2000) Exploring the phylogenetic structure of ecological communities: an example for rain forest trees. Am Nat 156: 145–155. 10856198 10.1086/303378

[2] Webb CO, Ackerly DD, McPeek MA, Donoghue MJ (2002) Phylogenies and community ecology. Annu Rev Ecol Syst 33: 475–505.

[3] Swenson NG, Enquist B, Pither J, Thompson J, Zimmerman JK (2006) The problem and promise of scale dependency in community phylogenetics. Ecology 87: 2418–2424. 17089650 10.1890/0012-9658(2006)87[2418:tpapos]2.0.co;2

[4] Mayfield MM, Levine JM (2010) Opposing effects of competitive exclusion on the phylogenetic structure of communities. Ecol Lett 13: 1085–1093. 10.1111/j.1461-0248.2010.01509.x 20576030

[5] Swenson NG (2013) The assembly of tropical tree communities—the advances and shortcomings of phylogenetic and functional trait analyses. Ecography 36: 264–276.

[6] Hardy OJ (2008) Testing the spatial phylogenetic structure of local communities: statistical performances of different null models and test statistics on a locally neutral community. J Ecol 96: 914–926.

[7] Kembel SW (2009) Disentangling niche and neutral influences on community assembly: assessing the performance of community phylogenetic structure tests. Ecol Lett 12: 949–960. 10.1111/j.1461-0248.2009.01354.x 19702749

[8] Kraft NJB, Cornwell WK, Webb CO, Ackerly DD (2007) Trait evolution, community assembly, and the phylogenetic structure of ecological communities. Am Nat 170: 271–283. 17874377 10.1086/519400

[9] González-Caro S, Parra JL, Graham CH, McGuire JA, Cadena CD (2012) Sensitivity of metrics of phylogenetic structure to scale, source of data and species pool of hummingbird assemblages along elevational gradients. PLoS One 7: e35472 10.1371/journal.pone.0035472 22558157 PMC3338702

[10] Swenson NG (2009) Phylogenetic resolution and quantifying the phylogenetic diversity and dispersion of communities. PLoS One 4: e4390 10.1371/journal.pone.0004390 19194509 PMC2633039

[11] Webb CO, Donoghue MJ (2005) Phylomatic: tree assembly for applied phylogenetics. Mol Ecol Notes 5: 181–183.

[12] Kress WJ, Erickson DL, Jones FA, Swenson NG, Perez R, Sanjur O, et al (2009) Plant DNA barcodes and a community phylogeny of a tropical forest dynamics plot in Panama. Proc Natl Acad Sci 106: 18621–18626. 10.1073/pnas.0909820106 19841276 PMC2763884

[13] Pei N, Lian J-Y, Erickson DL, Swenson NG, Kress WJ, Ye W-H, et al (2011) Exploring tree-habitat associations in a Chinese subtropical forest plot using a molecular phylogeny generated from DNA barcode loci. PLoS One 6: e21273 10.1371/journal.pone.0021273 21701680 PMC3119057

[14] Hebert PDN, Cywinska A, Ball SL, DeWaard JR (2003) Biological identifications through DNA barcodes. Proc R Soc B Biol Sci 270: 313–321. 10.1098/rspb.2002.2218 PMC1691236 12614582

[15] Lin C-P, Danforth BN (2004) How do insect nuclear and mitochondrial gene substitution patterns differ? Insights from Bayesian analyses of combined datasets. Mol Phylogenet Evol 30: 686–702. 15012948 10.1016/S1055-7903(03)00241-0

[16] Wilson JJ (2010) Assessing the value of DNA barcodes and other priority gene regions for molecular phylogenetics of Lepidoptera. PLoS One 5: e10525 10.1371/journal.pone.0010525 20479871 PMC2866325

[17] Blomberg SP, Garland T, Ives AR (2003) Testing for phylogenetic signal in comparative data: behavioral traits are more labile. Evolution 57: 717–745. 12778543 10.1111/j.0014-3820.2003.tb00285.x

[18] Pagel M (1999) Inferring the historical patterns of biological evolution. Nature 401: 877–884. 10553904 10.1038/44766

[19] Holzenthal RW, Blahnik RJ, Prather AL, Kjer KM (2007) Order Trichoptera Kirby, 1813 (Insecta), Caddisflies. Zootaxa 1668: 639–698.

[20] Kjer KM, Blahnik RJ, Holzenthal RW (2002) Phylogeny of caddisflies (Insecta, Trichoptera). Zool Scr 31: 83–91.

[21] Zhou X, Adamowicz SJ, Jacobus LM, Dewalt RE, Hebert PDN (2009) Towards a comprehensive barcode library for arctic life—Ephemeroptera, Plecoptera, and Trichoptera of Churchill, Manitoba, Canada. Front Zool 6: 30 10.1186/1742-9994-6-30 20003245 PMC2800108

[22] Zhou X, Jacobus LM, DeWalt RE, Adamowicz SJ, Hebert PDN (2010) Ephemeroptera, Plecoptera, and Trichoptera fauna of Churchill (Manitoba, Canada): insights into biodiversity patterns from DNA barcoding. J North Am Benthol Soc 29: 814–837.

[23] Ruiter D, Boyle EE, Zhou X (2013) DNA barcoding facilitates associations and diagnoses for Trichoptera larvae of the Churchill (Manitoba, Canada) area. BMC Ecol 13: 1–39. 10.1186/1472-6785-13-1 23425021 PMC3691766

[24] Ratnasingham S, Hebert PDN (2007) BOLD: the barcode of life data system (www.barcodinglife.org). Mol Ecol Notes 7: 355–364. 18784790 10.1111/j.1471-8286.2007.01678.x PMC1890991

[25] Boyle EE (2012) Community phylogenetics: methodological approaches and patterns in subarctic freshwater insect systems University of Guelph.

[26] Espeland M, Johanson KA (2010) The effect of environmental diversification on species diversification in New Caledonian caddisflies (Insecta: Trichoptera: Hydropsychidae). J Biogeogr 37: 879–890.

[27] Johanson KA, Malm T (2010) Testing the monophyly of Calocidae (Insecta: Trichoptera) based on multiple molecular data. Mol Phylogenet Evol 54: 535–541. 10.1016/j.ympev.2009.09.025 19786110

[28] Wiggins GB (1977) Larvae of the North American caddisfly genera (Trichoptera) Toronto, ON: University of Toronto Press.

[29] Ivanova NV, DeWaard JR, Hebert PDN (2006) An inexpensive, automation-friendly protocol for recovering high-quality DNA. Mol Ecol Notes 6: 998–1002.

[30] Ivanova NV, Grainger CM (2007) CCDB Protocols, COI amplification. Available: http://ccdb.ca//CCDB_DOCS/CCDB_Amplification.pdf

[31] Danforth BN, Fang J, Sipes S (2006) Analysis of family-level relationships in bees (Hymenoptera: Apiformes) using 28S and two previously unexplored nuclear genes: CAD and RNA polymerase II. Mol Phylogenet Evol 39: 358–372. 16412668 10.1016/j.ympev.2005.09.022

[32] Folmer O, Black M, Hoeh W, Lutz R, Vrijenhoek R (1994) DNA primers for amplification of mitochondrial cytochrome c oxidase subunit I from diverse metazoan invertebrates. Mol Mar Biol Biotechnol 3: 294–299. 7881515

[33] Hebert PDN, Penton EH, Burns JM, Janzen DH, Hallwachs W (2004) Ten species in one: DNA barcoding reveals cryptic species in the neotropical skipper butterfly *Astraptes fulgerator* . Proc Natl Acad Sci 101: 14812–14817. 15465915 10.1073/pnas.0406166101 PMC522015

[34] Kjer KM, Blahnik RJ, Holzenthal RW (2001) Phylogeny of Trichoptera (caddisflies): characterization of signal and noise within multiple datasets. Syst Biol 50: 781–816. 12116634 10.1080/106351501753462812

[35] Ivanova NV, Grainger CM (2007) CCDB Protocols, Sequencing. Available: http://ccdb.ca//CCDB_DOCS/CCDB_Sequencing.pdf

[36] Tamura K, Peterson D, Peterson N, Stecher G, Nei M, Kumar S (2011) MEGA5: molecular evolutionary genetics analysis using maximum likelihood, evolutionary distance, and maximum parsimony methods. Mol Biol Evol 28: 2731–2739. 10.1093/molbev/msr121 21546353 PMC3203626

[37] McKenna DD, Farrell BD (2010) 9-genes reinforce the phylogeny of Holometabola and yield alternate views on the phylogenetic placement of Strepsiptera. PLoS One 5: e11887 10.1371/journal.pone.0011887 20686704 PMC2912379

[38] Maddison WP, Maddison DR (2011) Mesquite: a modular system for evolutionary analysis.

[39] Nylander JAA (2004) MrModeltest v2 Program distributed by the author. Evolutionary Biology Centre, Uppsala University.

[40] Swofford DL (2002) PAUP*: phylogenetic analysis using parsimony. Version 4 Sinauer Associates, Sunderland, Massachusetts.

[41] Ronquist F, Teslenko M, van der Mark P, Ayres DL, Darling A, Höhna S, et al (2012) MrBayes 3.2: efficient Bayesian phylogenetic inference and model choice across a large model space. Syst Biol 61: 539–542. 10.1093/sysbio/sys029 22357727 PMC3329765

[42] Paradis E, Claude J, Strimmer K (2004) APE: analyses of phylogenetics and evolution in R language. Bioinformatics 20: 289–290. 14734327 10.1093/bioinformatics/btg412

[43] R Development Core Team (2008) R: a language and environment for statistical computing.

[44] Campbell V, Legendre P, Lapointe F-J (2011) The performance of the Congruence Among Distance Matrices (CADM) test in phylogenetic analysis. BMC Evol Biol 11: 64 10.1186/1471-2148-11-64 21388552 PMC3065422

[45] Kembel SW, Cowan PD, Helmus MR, Cornwell WK, Morlon H, Ackerly DD, et al (2010) Picante: R tools for integrating phylogenies and ecology. Bioinformatics 26: 1463–1464. 10.1093/bioinformatics/btq166 20395285

[46] Mitchell A, Mitter C, Regier JC (2000) More taxa or more characters revisited: combining data from nuclear protein-encoding genes for phylogenetic analyses of Noctuoidea (Insecta: Lepidoptera). Syst Biol 49: 202–224. 12118405

[47] Rokas A, Carroll SB (2005) More genes or more taxa? The relative contribution of gene number and taxon number to phylogenetic accuracy. Mol Biol Evol 22: 1337–1344. 15746014 10.1093/molbev/msi121

[48] Cummings MP, Otto SP, Wakeley J (1995) Sampling properties of DNA sequence data in phylogenetic analysis. Mol Biol Evol 12: 814–822. 7476127 10.1093/oxfordjournals.molbev.a040258

[49] Sagnes P, Mérigoux S, Péru N (2008) Hydraulic habitat use with respect to body size of aquatic insect larvae: case of six species from a French Mediterranean type stream. Limnologica 38: 23–33.

[50] Tolonen KT, Hämäläinen H, Holopainen IJ, Mikkonen K, Karjalainen J (2003) Body size and substrate association of littoral insects in relation to vegetation structure. Hydrobiologia 499: 179–190.

[51] Freckleton RP, Harvey PH, Pagel M (2002) Phylogenetic analysis and comparative data: a test and review of evidence. Am Nat 160: 712–726. 10.1086/343873 18707460

[52] Harmon LJ, Weir JT, Brock CD, Glor RE, Challenger W (2008) GEIGER: investigating evolutionary radiations. Bioinformatics 24: 129–131. 18006550 10.1093/bioinformatics/btm538

[53] Münkemüller T, Lavergne S, Bzeznik B, Dray S, Jombart T, Schiffers K, et al (2012) How to measure and test phylogenetic signal. Methods Ecol Evol 3: 743–756.

[54] Sólymos P, Lele SR (2012) Global pattern and local variation in species-area relationships. Glob Ecol Biogeogr 21: 109–120.

[55] Talavera G, Vila R (2011) What is the phylogenetic signal limit from mitogenomes? The reconciliation between mitochondrial and nuclear data in the Insecta class phylogeny. BMC Evol Biol 11: 315 10.1186/1471-2148-11-315 22032248 PMC3213125

[56] Bergsten J, Bilton DT, Fujisawa T, Elliott M, Monaghan MT, Balke M, et al (2012) The effect of geographical scale of sampling on DNA barcoding. Syst Biol 61: 851–869. 22398121 10.1093/sysbio/sys037 PMC3417044

