# Supplementary figures and images for: Community Phylogenetics: Assessing Tree Reconstruction Methods and the Utility of DNA Barcodes
Source: PLoS One. 2015 Jun 25;10(6):e0126662. doi: 10.1371/journal.pone.0126662 (PMC4481530; doi:10.1371/journal.pone.0126662)

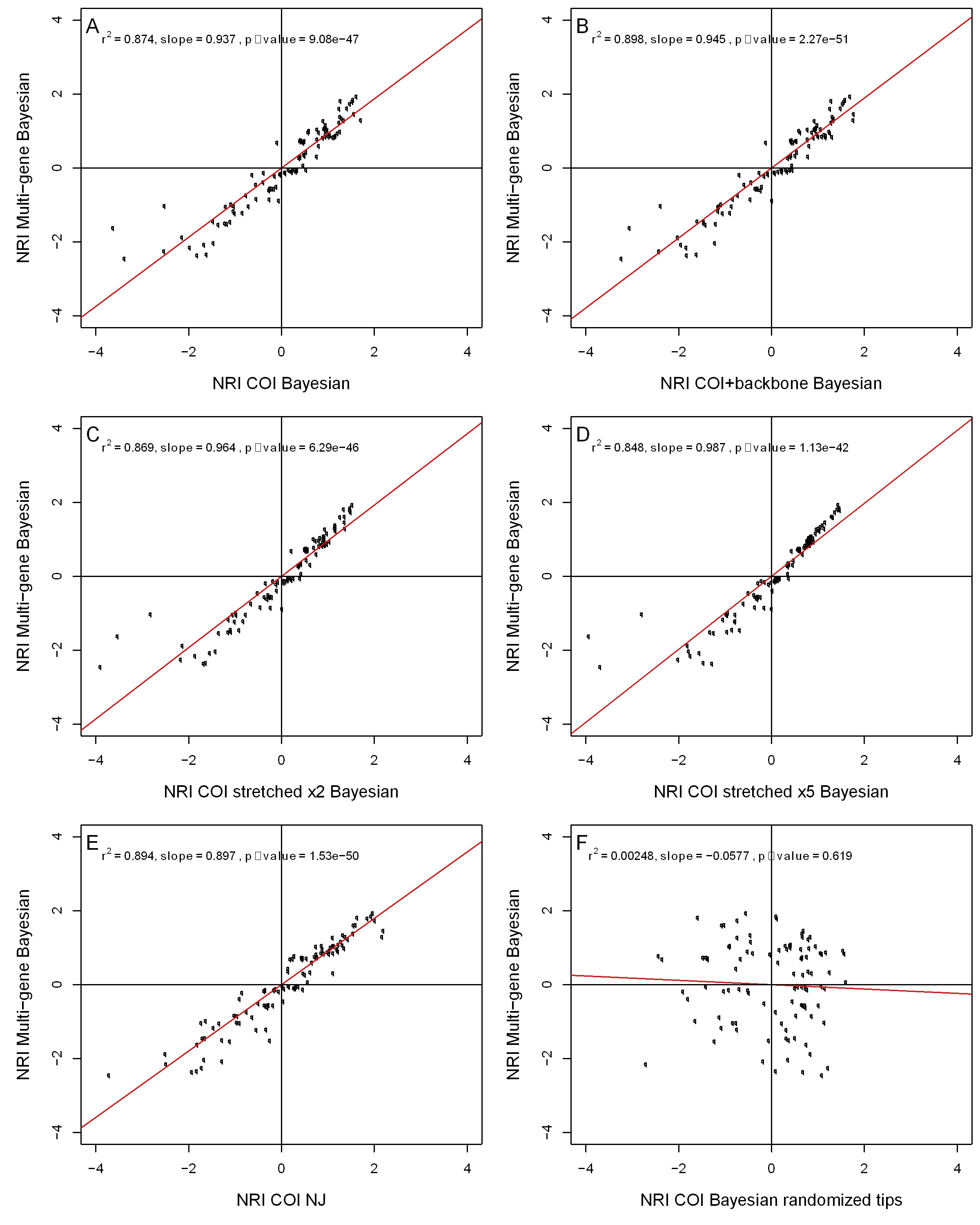

Supplement: S1 Fig — Red lines show the equation for each linear model, which is forced through 0. (TIFF) [file pone.0126662.s001.tiff]

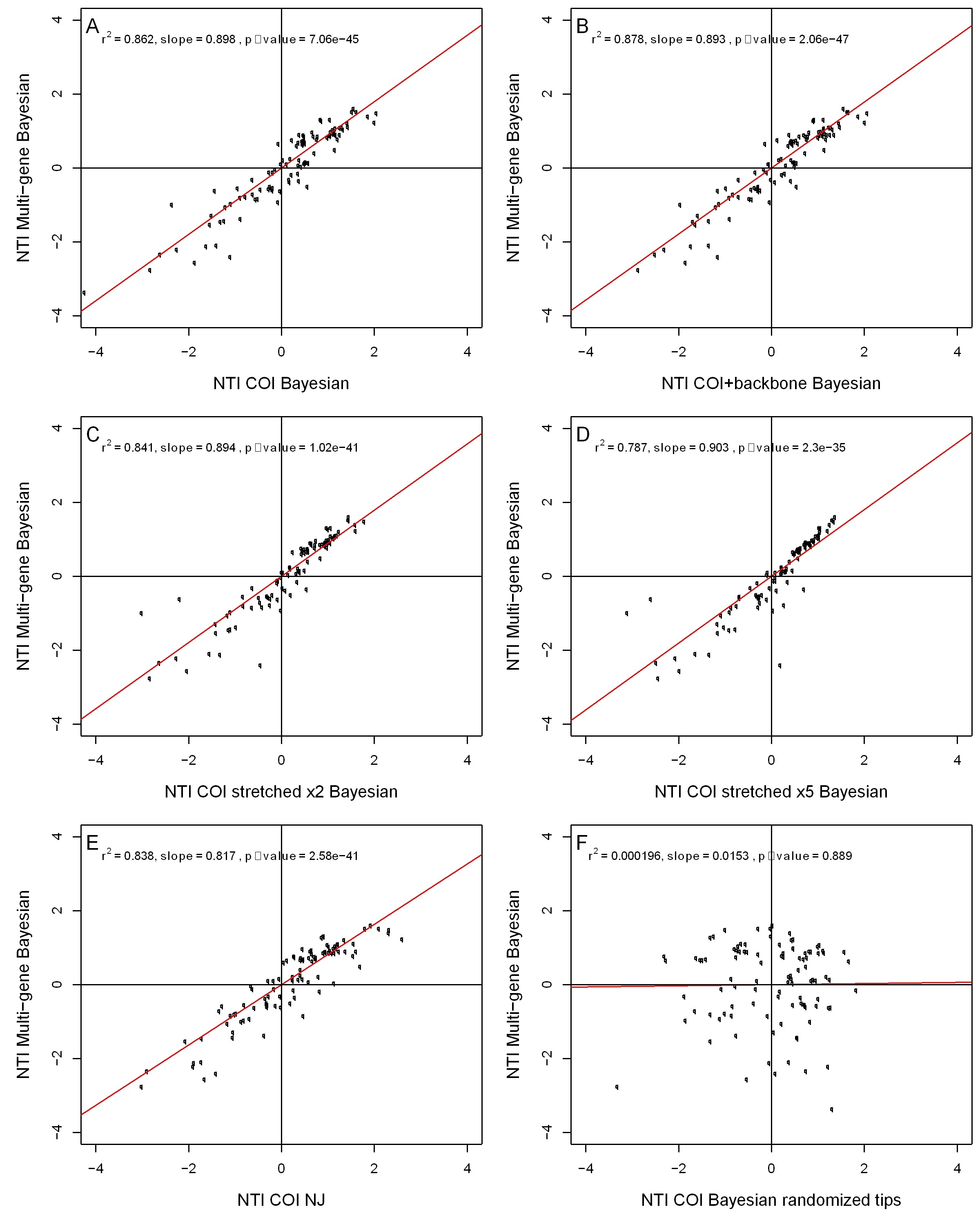

Supplement: S2 Fig — Red lines show the equation for each linear model, which is forced through 0. (TIFF) [file pone.0126662.s002.tiff]
